# The role of innate immune system in respiratory viral infection related asthma

**DOI:** 10.3389/fcimb.2025.1604831

**Published:** 2025-06-25

**Authors:** Xiao Wu, Feifei Huang, Wenbo Yao, Zheng Xue

**Affiliations:** ^1^ Shanghai Municipal Hospital of Traditional Chinese Medicine, Shanghai University of Traditional Chinese Medicine, Shanghai, China; ^2^ Shanghai Children’s Hospital, School of Medicine, Shanghai Jiao Tong University, Shanghai, China; ^3^ Key Laboratory of Organ Regeneration and Transplantation of Ministry of Education, Institute of Immunology, The First Hospital, Jilin University, Changchun, China

**Keywords:** asthma, viral infection, innate immune cells, cytokines, recurrent asthma attacks

## Abstract

The association between viral infections and asthma has garnered significant attention in recent years. Accumulating evidence suggests that viral infections can trigger asthma exacerbations, while asthma may also influence the susceptibility to viral infections, thereby creating a cycle of worsening symptoms and recurrent asthma attacks. Given that asthma is predominantly driven by Type 2 immune responses and viral infections are typically associated with Type 1 immune responses, the innate immune cells and cytokines that participate in both conditions appear to be the critical bridge connecting these two processes. In particular, innate immune cells play a pivotal role in modulating the immune response at the interface of viral infections and asthma. In this review, we summarize the key innate immune cells and cytokines involved in viral infections and asthma, highlighting their immunoregulatory mechanisms. We aim to provide novel perspectives and potential therapeutic directions for the clinical management of recurrent asthma attacks induced by viral infections.

## 1 Introduction

Asthma, a pervasive chronic inflammatory condition of the airways, stands as one of the most prevalent global health challenges. It is marked by an excessive response of the bronchi to diverse stimuli, manifesting in recurrent episodes of wheezing, respiratory distress, and coughing, all of which are intricately linked to reversible airway obstruction (Papi et al., 2018). Asthma affects more than 300 million people around the world, and unfortunately, the number of individuals suffering from this ailment is on the rise (Schleich et al., 2023). Patients with severe, persistent asthma, comprising merely 5% to 10% of the asthma patient population, disproportionately contribute to the overall incidence of asthma and escalate healthcare costs, thereby placing a substantial economic strain on society (Lang, 2015).

Respiratory viral infections emerge as a significant trigger for asthma. The majority of acute exacerbations of asthma, especially in children, coincide with respiratory viral infections (Johnston and Sears, 2006). RV remains the most potent objective risk factor for wheezing in young children and school-aged asthma, exhibiting an odds ratio (OR) of 45, contingent upon auxiliary factors like sensitization to aeroallergens (Rubner et al., 2017). Furthermore, RV also serves as a significant contributor to adult asthma, accounting for as much as 83% of cases (Papadopoulos et al., 2011). In addition to RV, respiratory syncytial virus (RSV) is also a major pathogen that triggers asthma, with a detection rate of 50%-80% in hospitalized cases (Jartti et al., 2020). a cohort study indicated that preventing RSV infection during infancy could reduce the proportion of current asthma at the age of 5 by about 15% (Rosas-Salazar et al., 2023). Similarly, individuals infected with coronavirus disease (COVID-19) in South Korea have an incidence rate of new-onset asthma that is 2.1 times higher than those who have not been infected (Kim et al., 2024). Additionally, influenza virus, parainfluenza virus, adenovirus, and coronavirus may also be associated with the development of asthma (Ha et al., 2024).

In addition to being a trigger for asthma attacks, viral infections can also lead to exacerbations and recurrent episodes of asthma. Influenza virus appears to be a key factor in triggering severe exacerbations of asthma among adults (Papadopoulos et al., 2011). Besides, The association between RV and COVID-19 and the risk of asthma exacerbation is strongly correlated (Johnston et al., 1996; Johnston et al., 2006; Tydeman et al., 2023). Furthermore, although coronaviruses, human metapneumoviruses, parainfluenza viruses, adenoviruses, and bocaviruses have been detected in association with asthma exacerbations, their occurrence is relatively low (Yoo et al., 2013).

It Is well-known that Th2-type immune responses predominate in asthma, whereas viral infections mainly induce Th1-type immune responses. Recent advances in our understanding of the sentinel role played by innate immunity provides new targets for disease prevention and treatment. These include pathways of innate stimulation by environmental or endogenous pathogen-associated molecular patterns (PAMPs) and danger-associated molecular patterns (DAMPs) to influence the activation and trafficking of DCs, innate sources of cytokines, and the identification of new T cell subsets and lymphoid cells.

The aim of this article is to provide a comprehensive review of the immune mechanisms underpinning viral infection’s role in asthma onset and exacerbation, examining the intricate interplay between immune cells, cytokines, and viral infections, while also exploring viable treatment avenues.

## 2 The pathogenesis of asthma induced by viral infections

The innate immune system constitutes the primary defensive response to pathogen incursion and is capable of swiftly recognizing and responding to a diverse array of pathogens, including viruses, bacteria, and fungi. This rapid response is pivotal in controlling the initial dissemination of infections. Within the context of asthma pathogenesis, the innate immune system assumes a central role. It discerns environmental allergens through pattern recognition receptors, subsequently activating dendritic cells and macrophages. These cells then secrete inflammatory mediators such as IL-33 and TSLP. These alarmins further induce the differentiation and activation of Th2 cells, culminating in the production of Th2 cytokines, including IL-4, IL-5, and IL-13. This process intensifies airway inflammation and leads to the characteristic features of asthma, namely airway hyperresponsiveness and inflammation (Pivniouk et al., 2020).

It is well known that early-life respiratory viral infections are intimately linked to the onset of asthma and the main types of viruses are RSV and RV (Kusel et al., 2007; Ruotsalainen et al., 2013). The infection of RSV or RV causes bronchiolitis in varying age cohorts, and infants admitted to hospitals due to bronchiolitis face an elevated risk of subsequently developing asthma (Jartti et al., 2020; Nanishi et al., 2022; Zhu et al., 2022). The elevated levels of CCL5 in nasal secretions subsequent to RSV infection is linked to the progression of virus-induced bronchiolitis (Hizawa, 2023). The overproduction of TSLP triggered by RV16 or double-stranded RNA (dsRNA) in bronchial epithelial cells (BECs) and primary human nasal epithelial cells (HNECs) may be associated with the onset of asthma (Xu et al., 2010; Calvén et al., 2012). Besides RV and RSV, infection by the influenza A virus triggers the NLRP3 inflammasome, resulting in augmented production of IL-33 by alveolar macrophages. This subsequently activates innate lymphoid cell types, leading to the secretion of substantial quantities of IL-13, which in turn causes AHR (Chang et al., 2011) (**Table 1**). The reprogramming of airway epithelial cells during the developmental phase subsequent to a viral infection, resulting in their malfunction, manifested by heightened barrier permeability and diminished antiviral response, among other factors (Connelly et al., 2021; Berdnikovs et al., 2024a; Berdnikovs et al., 2024b).

**Table 1:** Respiratory virus-associated receptors and cytokines.

Virus	Type	Subgroups	Receptor	Downstream-related cytokines
RSV	RNA	A, B	IGF1R,NCL	CCL5,TSLP,TGF-β,IL-1β,IL-5,IL-9,IL-13,IL-33,NMUR1,MMP-12
RV	RNA	A, B, C	RV-A:ICAM-1&LDL-R RV-B:ICAM-1 RV-C:CDHR3	ICAM-1,CXCL1,CCL5,CXCL9,TSLP,TGF-β,IL-1β,IL-5,IL-9,IL-33,IFN-λ
IAV	RNA	A, B, C	Sialic Acid Receptor,mGluR2	ICAM-1,TGF-β,IL-5,IL-33,IFN-α,IFN-β,NMU

RSV, respiratory syncytial virus; RV, rhinoviruses; IAV, influenza virus A.

Patients suffering from asthma may encounter exacerbated and recurrent asthma attacks following respiratory viral infections. After RV virus infection, epithelial cells of asthmatic patients can increase the production of various type 1 cytokines, including granulocyte colony-stimulating factor, Interferon (IFN) -γ, and tumor necrosis factor (TNF) -α, as well as type 2 cytokines such as IL-4, IL-5, and IL-13, thereby inducing the exacerbation of asthma (Thomas et al., 2009; Muehling et al., 2020). Excessive levels of TGF-β in the airways of asthma patients can promote enhanced viral replication and pro-inflammatory responses after RV infection, which may stem from its immunosuppressive effects via actions on IFN regulatory factor-3 pathways (Thomas et al., 2009). A genome-wide association study identifies cadherin related family member 3 (CDHR3) as a susceptibility locus for severe early childhood asthma exacerbation and CDHR3 can mediate RV-C entry into host cells, thereby causing wheezing diseases and exacerbation of asthma (Bønnelykke et al., 2014; Bochkov et al., 2015). This may be due to increased IL-17A production during viral infections (Eliasen et al., 2022).

While numerous studies have pinpointed various connections between respiratory viral infections and asthma, the precise mechanisms underlying these associations remain to be elucidated. In this review, we provide a summary and delve into the innate immune system between respiratory viruses infection and asthma from the perspective of different immune cells and cytokines.

## 3 The immunoregulatory role of innate immune cells in infection-induced asthma.

Innate immune cells are pivotal in the development of asthma and viral infections. They serve as the initial defense against viral pathogens, primarily through cells such as monocytes/macrophages, neutrophils, and natural killer (NK) cells. In atopic asthma, eosinophils are the key innate immune cells involved in the type 2 immune response, whereas neutrophils predominantly participate in the immune response in non-atopic asthma. Furthermore, ILC2 have also been found to play a significant role in immune regulation in asthma. We will delve into the relationship between asthma and viral infections by examining the roles of various innate immune cells.

### 3.1 Eosinophil

In addition to clinical typing of asthma, other phenotypes are based on trigger factors. Based on the infiltration of inflammatory cells in induced sputum, asthma can be categorized into four distinct inflammatory phenotypes: eosinophilic asthma, neutrophilic asthma, mixed granulocytic asthma, and paucigranulocytic asthma, with eosinophilic asthma comprising approximately 50% of all cases (Simpson et al., 2006).

Eosinophils are pivotal in the pathogenesis of asthma. The development of eosinophils is regulated by cytokines such as IL-5 and GM-CSF, which promote the proliferative properties of inflammatory-like and lung-resident-like eosinophils in the blood of asthma patients (Palacionyte et al., 2022). Mature eosinophils are recruited to the sites of airway inflammation through interactions with adhesion molecules on endothelial cells under the influence of chemokines (McBrien and Menzies-Gow, 2017). Once in the airway tissue, eosinophils are activated and release a variety of mediators and cytokines, including eosinophil cationic protein (ECP), eosinophil peroxidase (EPO), major basic protein (MBP), IL-4, IL-5, and IL-13 (Hussain and Liu, 2024). These mediators and cytokines act in concert to induce airway inflammation and tissue damage. Toxic granule proteins such as ECP, EPO, and MBP directly damage airway epithelial cells, thereby disrupting the airway epithelial barrier function and further exacerbating the inflammatory response. Cytokines such as IL-4 and IL-13 increase the contractility of airway smooth muscle cells, rendering them more sensitive to stimuli. Moreover, these mediators also promote the hypertrophy and proliferation of airway smooth muscle cells, leading to airway remodeling, which is characterized by thickening of the airway wall, thickening of the basement membrane, fibrosis, and hyperplasia of mucus glands (McBrien and Menzies-Gow, 2017).While eosinophilic inflammation has been associated with virus-induced asthma exacerbations (Bjerregaard et al., 2017), whether it is a direct cause remains to be investigated. Multiple mechanistic pathways suggest eosinophils may contribute to exacerbation severity during viral infections. The presence of eosinophilic inflammation could potentially serve as a risk factor for the aggravation of virus-associated asthma, particularly in the setting of RV infections (Mathur et al., 2013). Human airway epithelial cells produce IFN-γ inducible protein-10 (IP-10)/C-X-C motif chemokine (CXCL) 10 *in vitro* and *in vivo* upon rhinovirus infection (Tan et al., 2018; Muehling et al., 2022), The release of IP-10 is closely associated with acute virus-induced asthma. IP-10 and its receptor CXCR3 may play a significant role in exacerbating acute rhinovirus-induced asthma, representing potential therapeutic targets (Wark et al., 2007). It could be due to the role of IP-10 as an activator for eosinophils via β2 integrin and CXCR3, leading to eosinophil adherence, O−2 formation, and the excretion of eosinophil-derived neurotoxin *in vitro* (Takaku et al., 2011).The exacerbation of asthma induced by rhinovirus has been shown to be associated with the upregulation of intercellular adhesion molecule-1 (ICAM-1) in the epithelium (Grünberg et al., 2000), which may be due to increased adhesiveness between eosinophils and epithelial cells mediated by ICAM-1 binding to leukocyte function associated antigen-1 (LFA-1) and macrophage-1 antigen (Mac-1) (Wardlaw et al., 1994).

### 3.2 Neutrophils

Non-eosinophilic asthma is primarily characterized by the presence of neutrophils and can be triggered by exposure to bacterial endotoxins and viral infections (Douwes et al., 2002). Acute asthma attack induced by viral infections are intimately linked to increasing neutrophilic inflammation (Wark et al., 2002). The infections caused by both RSV and influenza virus A (IAV) are associated with the formation of neutrophil extracellular traps (NETs), which is an innate immune response of host to capture pathogens and exert an antiviral effect (Cortjens et al., 2018; Stacey et al., 2021). Whereas the abundant formation of NETs may cause airway remodeling and excessive mucus secretion, leading to airway occlusion and triggering asthma (Brinkmann et al., 2004; Saitoh et al., 2012; Cheng and Palaniyar, 2013; Liu et al., 2017). In the Sendai virus (Sev) -induced asthma mouse model, effectively managing the activation of neutrophils and the subsequent formation of NETs holds the potential to alleviate acute episodes and potentially forestall subsequent airway remodeling (Akk et al., 2016). In addition, NETs promoted fluorescently labelled HDM extract uptake by lung CD11bLy-6C dendritic cells (DCs) and mediated allergic airway inflammation triggered by flu infection (Radermecker et al., 2019). It is evident that neutrophils play a pivotal role in the pathogenic mechanism of virus-induced asthma.

### 3.3 ILC2

ILC2s cells are type 2 innate lymphoid cells which respond to epithelial cell-derived cytokines such as IL-25, IL-33 and TSLP (Moro et al., 2015). ILC2s mainly play a role in the induction of eosinophilic inflammation in asthma patients through IL-5, IL-13, IL-33, and TSLP, promoting the onset and the exacerbation of asthma, emphasizing the therapeutic potential of targeting ILC2 (Smith et al., 2016; Matsuyama et al., 2022).ILC2s that are induced upon influenza viral infections may contribute to exacerbations of airway inflammation upon allergen exposure (Li et al., 2019). Furthermore, research has found that memory ILC2s are ILC2s that acquire immune memory and play a critical role in exacerbating virus-induced asthma. Following infections of RSV, ILC2s are activated by epithelial-derived alarmins including IL-33 and TSLP, while a subset differentiates into mILC2s with long-term persistence (Martinez-Gonzalez et al., 2016; Stier et al., 2016). These mILC2s exhibit elevated expression of IL-17RB which is IL-25 receptor and transcription factors TCF-1 and TOX, which are essential for memory formation and migration. During asthma remission, mILC2s reside in the small intestine lamina propria via CCR9/CCL25 signaling, but upon viral rechallenge, they migrate to the lungs through S1P/S1PR1-mediated circulation, rapidly producing IL-13 to amplify airway inflammation (Bao et al., 2024). Transcriptomic analyses reveal mILC2s maintain “primed” cytokine programs like IL-5/IL-13 mRNA and upregulate detoxification pathways such as GSTs, conferring steroid resistance (Verma et al., 2021). In humans, circulating CD45RO^+^ mILC2s correlate with asthma severity and poor response to corticosteroids, highlighting their role in virus-driven asthma exacerbations (van der Ploeg et al., 2021; Bao et al., 2024). Thus, mILC2s bridge innate immunity and allergic memory, linking viral infections to chronic asthma progression through enhanced responsiveness and inter-organ trafficking.

### 3.4 NK

NK cells are renowned for their antiviral capabilities, yet the precise mechanisms underlying their functioning in asthma remain elusive remain elusive and may exhibit a dual nature, potentially contributing to either the promotion or inhibition of the disease (Erten et al., 2008; Vivier et al., 2008). Studies using different experimental models have attempted to elucidate the role of NK cells in asthma pathogenesis. For instance, Lunding et al. (2015) employed an OVA/alum-induced murine asthma model with poly I/C administration to mimic viral exacerbations, demonstrating that NK cell depletion impaired the development of asthma-like features in this system (Verma et al., 2021). While this suggests NK cells may contribute to asthma pathogenesis in certain experimental conditions, it should be noted that this model did not involve actual viral infection. However, it has been reported that there is an upregulated proportion of peripheral NK cells in asthma patients subsequent to viral upper respiratory infections (Liu et al., 2023). The heightened levels of peripheral CD3^-^CD56^+^CD16^+^ NK cells observed in asthma patients subsequent to viral upper respiratory infections are positively associated with a dominant Th1-like immune response during asthma episodes. This finding underscores the protective role of NK cells to enhance antiviral Th1 immunity, presenting a viable therapeutic avenue for mitigating infection-triggered asthma exacerbations (Liu et al., 2023).

### 3.5 MDSC

MDSC is a group of heterogeneous immature myeloid cells with powerful immunosuppressive capabilities (Hegde et al., 2021). Recent studies have highlighted the increasingly intricate and pivotal role of myeloid-derived suppressor cells (MDSCs) in various pulmonary diseases, including infectious lung disorders, pulmonary arterial hypertension, and lung cancer (Kolahian et al., 2016; Ma et al., 2018; Zhang H. et al., 2023; Zhang MN. et al., 2023). Recent preclinical studies using murine models have provided insights into the role of MDSCs in asthma exacerbations. Notably, it has been demonstrated in a mouse model of influenza A virus (IAV)-induced asthma exacerbation that pulmonary MDSCs, particularly the monocytic subset (M-MDSCs), exhibited both increased abundance and enhanced immunosuppressive activity, which correlated with inhibited T cell proliferation and attenuated respiratory symptoms (van Geffen et al., 2024).

While these murine studies suggest a potential protective role for MDSCs in virus-induced asthma exacerbations, it should be emphasized that direct evidence from human studies remains limited. Further investigation is required to determine whether similar mechanisms operate in human asthma pathophysiology.

### 3.6 Pulmonary macrophages

Pulmonary macrophages, as the central regulators of lung immunity, primarily consist of alveolar macrophages (AMs) and interstitial macrophages (IMs), which exhibit distinct localization, functional roles, and disease-modulating capabilities. AMs reside in the alveolar lumen, directly exposed to the external environment, and possess self-renewal capacity, whereas IMs are distributed in the lung interstitium and remain relatively scarce under steady-state conditions. However, during inflammation, circulating monocytes can migrate into the lungs and differentiate into IMs (Shi et al., 2021). Research has demonstrated that these macrophage subsets contribute to pulmonary immune homeostasis and disease progression through divergent mechanisms.

AMs serve as the first line of defense in the lungs, responsible for capturing, ingesting, and neutralizing inhaled pathogens and particulate matter (Aegerter et al., 2022). These cells originate from the yolk sac during embryonic development, reside in the alveoli, and self-renew throughout life without reliance on bone marrow contributions. In response to inflammatory stimuli, monocytes from the bone marrow are recruited to the lungs and differentiate into alveolar macrophages (Hu and Christman, 2019). During acute lung injury, AMs act as the primary coordinators of the inflammatory response, initiating and modulating inflammation through the secretion of pro-inflammatory cytokines and chemokines (such as IL-6, IL-8, or CXCL10), and responding to viral infections by activating type I interferon signaling and enhancing the expression of pattern recognition receptors (Malainou et al., 2023). AMs play a significant role in the onset and exacerbation of virus-induced asthma. In young mice infected with Sendai virus (SeV), AMs exhibit a more pronounced type II inflammatory response, leading to pathologic features of asthma, whereas AMs in adult mice may generating a low-inflammatory state post-infection (Hazan et al., 2022). In a mouse model of HDM-induced allergic airway inflammation, RSV infection induces alveolar macrophages to produce high levels of MMP-12, thereby increasing airway hyperresponsiveness (AHR) and the accumulation of inflammatory cells in the airways, exacerbating asthma (Makino et al., 2021). MuHV-4 infection mitigates the type 2 immune response to house dust mites in mice, thereby exerting a protective effect against the development of asthma, through the reprogramming of alveolar macrophages and the reduction of the number and function of ILC2s (Loos et al., 2023). Furthermore, the number and functionality of AMs differ between young and adult mice, and these differences may have significant implications for the development and resolution of asthma and other respiratory diseases (Hazan et al., 2022).

IMs are a type of resident immune cells located in the interstitial region between the alveolar epithelium and capillaries, and they possess unique immune regulatory functions distinct from those of AMs. Studies have shown that IMs highly express MHC-II molecules and have antigen-presenting capabilities similar to dendritic cells. They are able to take up and process soluble antigens, such as OVA, and subsequently induce the activation and differentiation of CD4^+^ T cells into Foxp3^+^ regulatory T cells (Tregs), a process that is mediated by the IL-10 and TGF-β signaling pathways (Legrand et al., 2024). During the pathogenesis of asthma, IMs play a crucial protective role through their anti-inflammatory properties. In a HDM-induced asthma model, IMs are the primary source of IL-10, and they significantly alleviate neutrophil-dominated airway inflammation by inhibiting the expression of Th2/Th17-related inflammatory cytokines such as IL-13, IL-17, GM-CSF, and TNF-α (Kawano et al., 2016). In asthma exacerbations triggered by viral infections, IMs also exert regulatory functions. A mouse model study demonstrated that influenza virus infection can lead to elevated levels of Th2 cytokines such as IL-4 and IL-5, along with a significant downregulation of IL-10, resulting in intense inflammation in the airway mucosa and interstitial regions (Nazir et al., 2008). This suggests that impaired function of anti-inflammatory IMs is one of the important mechanisms underlying asthma exacerbation. Moreover, in neutrophilic asthma, a phenotype of asthma that is unresponsive to glucocorticoids, a regulatory CD39^+^CD9^+^ IMs subpopulation has been identified. In an IL-23/Th17-driven neutrophilic inflammation model, this subpopulation can bind to neutrophils in a CD9-dependent manner and degrade ATP via CD39 to inhibit the formation of NETosis and the activation of Th17 cells, thereby alleviating airway inflammation. Clinical samples have also shown a significant reduction of CD39^+^CD9^+^ IMs in patients with severe asthma, indicating their potential as therapeutic targets (Han et al., 2024). In a mouse model of virus-induced asthma exacerbation using HDM and poly(I:C), the non-antibacterial macrolide EM900 reduced the recruitment and percentage of interstitial macrophages in lung tissue and suppressed the production of cytokines such as IL-6, RANTES, and MIP-2 in macrophages, thereby alleviating airway inflammation. These findings suggest that EM900 may be a potential therapeutic option for asthma, particularly for virus-induced exacerbations (Sadamatsu et al., 2020).

### 3.7 Dendritic cells

Dendritic cells (DCs) orchestrate the interplay between viral infection and asthma exacerbation by integrating innate antiviral responses and adaptive allergic immunity. Type I conventional DCs (cDC1), specialized in cross-presenting viral antigens to CD8^+^ T cells and producing type I interferons (IFNs), are significantly reduced in the lower airways of asthmatics, correlating with increased viral replication, eosinophilic inflammation, and impaired lung function during rhinovirus infection (van Rijt et al., 2005; Cameron et al., 2022). This deficiency is linked to atopic status, as baseline cDC1 numbers inversely correlate with serum IgE levels and house dust mite-specific IgE, highlighting a critical role in antiviral defense (Cameron et al., 2022). Meanwhile, plasmacytoid DCs (pDCs), the primary source of type I IFNs, exhibit elevated expression of the high-affinity IgE receptor (FcϵRIα) in asthmatics, which suppresses IFN-α production and exacerbates viral persistence (Xi and Upham, 2020). During RSV viral infection, myeloid DCs activated by RSV-infected epithelial cells upregulate co-stimulatory molecules CD86 and OX40L and secrete IL-13, driving CD4^+^ T cell differentiation into Th2 cells (Lambrecht and Hammad, 2012). Additionally, DCs can induce a unique subset of Th17/Th2 hybrid cells that co-express RORγt and GATA-3, producing both IL-17 and IL-13 to amplify airway hyperreactivity and mucus hypersecretion (Raymond et al., 2011). Additionally, DCs from asthmatics show impaired migration and antigen-presenting capacity, particularly in priming antiviral CD8^+^ T cells, which is associated with reduced cDC1 numbers and exacerbated viral burden (Cameron et al., 2022). Thus, DCs act as critical orchestrators, linking viral detection to aberrant type 2 inflammation and chronic asthma progression through impaired antiviral immunity and augmented Th2/Th17 responses.

## 4 The immunoregulatory role of cytokines in infection and asthma

Cytokines, which include small molecular peptides or glycoproteins, are a category of substances with diverse active functions. They are primarily synthesized and secreted by the body’s innate and adaptive immune cells. As the most direct executors of immune responses, cytokines regulate the interactions among immune system cell populations and between the immune system and other cell types. Monitoring changes in cytokine levels is the most direct and rapid method for assessing the immune status of clinical patients, evaluating treatment outcomes, predicting prognosis, and identifying risks. It is also crucial for the rational and precise administration of medication.

### 4.1 Interleukin IL33/IL9/IL-1β/IL5/IL4

Type 2 cytokines play a key role in the development of asthma. IL-33 is a member of the IL-1 family. IL-33 induces signals through its receptor IL-1RL1 in various immune and structural cells, leading to the production of type 2 cytokines and chemokines, thereby driving type 2 responses (Saikumar Jayalatha et al., 2021). IL-33 may promote the exacerbation of virus-induced asthma by enhancing type 2 inflammation or by attenuating innate and adaptive Th1-like and cytotoxic responses (Jackson et al., 2014; Ravanetti et al., 2019). IAV infection has been shown to increase IL-33, a process that may be achieved through Toll interacting protein (Tollip), suppressing IL-33 signaling can alleviate excessive airway type 2 inflammation in human subjects with IAV infection (Nouri et al., 2023; Schaunaman et al., 2023). IL-33 induces the expression of antiviral genes in mast cells (MCs), but at the same time increases the susceptibility of these cells to human rhinovirus (HRV) by upregulating ICAM1 (the primary receptor for HRV) and low-density lipoprotein (LDLR) (a secondary receptor for HRV entry into cells) (Akoto et al., 2022). IL-9 is produced by CD4^+^ Th2 and Th9 subsets, as well as by ILC2s (Doherty and Broide, 2022). In an asthma exacerbation model induced by poly I:C, the reduction of inflammatory mediators such as IL-13 and IL-9 can significantly alleviate airway inflammation and hyperresponsiveness (Ujino et al., 2017). In patients with recurrent wheezing induced by RSV and HRV, the expression level of IL-9 are higher (Sugai et al., 2016).Virus-induced asthma attacks are characterized by an increase in Th1-type neutrophils and Th2-type inflammation, which is associated with the secretion of IL-1β. Inhibiting IL-1β during RSV infection can improve RSV immunopathology and reduce the consequences of allergen-induced asthma, making it a potential new therapeutic target for reducing early virus-induced asthma development (Schuler et al., 2020). IL-1β induces neutrophil inflammation and may also increase the expression of Th2-type cytokines, exacerbating asthma deterioration (Mahmutovic Persson et al., 2018). IL-5 exacerbates airway inflammation by activating and recruiting eosinophils and may directly participate in the process of airway remodeling (AbuJabal et al., 2024). In asthma patients infected with RV, IL-5 inhibits the function of pDCs and the expression of TLR7 by promoting the maturation and survival of eosinophils, thereby suppressing the antiviral response and leading to the exacerbation of asthma (Hatchwell et al., 2015; Dill-McFarland et al., 2022). As key cytokines of Th2-type inflammation, IL-4 and IL-13 play a crucial role in the pathogenesis of asthma. These two cytokines are closely related, sharing a similar structure and a common receptor subunit (IL-4Rα) (Bernstein et al., 2023). *In vitro* studies have shown that IL-4 and IL-13 can significantly enhance the contractile response of human small airways to histamine, LTD4, and carbachol. They also cause a marked increase in calcium ion influx in airway smooth muscle cells. These actions make them important cytokines in the development of airway hyperresponsiveness (Oh et al., 2010). In addition, IL-4 can increase the permeability of airway epithelial cells, promote the activation of fibroblasts and the deposition of extracellular matrix (ECM), and enhance the sensitivity of smooth muscle cells to histamine. Thus, it plays a key role in airway remodeling (Sahnoon et al., 2025).

In research on the relationship between viral infections and asthma, it has been observed that in HDM-sensitized mice infected with H1N1, the levels of IL-4 and IL-5 in bronchoalveolar lavage fluid (BALF) significantly increase at multiple time points following infection, while the level of the Th1 inflammatory mediator IFN-γ decreases. Blocking IL-4Rα can reduce weight loss and viral load in HDM-sensitized mice (Shahangian et al., 2021). In the case of rhinovirus (RV) infection, which induces the production of pro-inflammatory cytokines by macrophages, IL-4 promotes the polarization of macrophages towards the M2 phenotype, altering their response to RV infection. This finding may offer new insights into the mechanisms underlying RV-induced asthma exacerbations (Saba et al., 2014). Children who have suffered from RSV bronchiolitis have been found to have a significantly higher frequency of IL-4 - producing T cells in response to cat allergens(Feld), which is closely associated with an increased risk of asthma and wheezing (Pala et al., 2002).

### 4.2 TSLP

TSLP is a cytokine derived from epithelial cells that plays a pivotal role in the development of asthma and various allergic conditions. In allergic asthma, TSLP triggers downstream signaling pathways upon binding to its unique receptor, TSLPR. This interaction facilitates the maturation of DCs and the differentiation of Th2 cells, leading to an increased production of inflammatory cytokines. Consequently, this process amplifies airway inflammatory responses (Al-Shami et al., 2005; Divekar and Kita, 2015). In virus-induced asthma, the role of TSLP is particularly important. Respiratory viral infections can induce high levels of TSLP production in airway epithelial cells, which not only promotes the recruitment and activation of inflammatory cells, leading to the onset of asthma, but may also exacerbate the acute exacerbation of asthma by affecting the secondary response of memory CD8^+^ T cells (O’Sullivan et al., 2001; Gilliet et al., 2003; Yadava et al., 2013). During RSV infection, the activation of the uric acid pathway increases the expression of TSLP, which in turn activates ILC2s to produce IL-13, promoting a Th2-type immune response associated with the development of asthma (Fonseca et al., 2020). In influenza-induced exacerbation of asthma, TSLP may promote the accumulation of Cullin5 in alveolar macrophages, which is involved in various cellular processes including cell migration, DNA damage repair, and inflammation, thereby inhibiting antiviral immune responses and promoting neutrophilic inflammation, leading to worsening of asthma symptoms (Zhang et al., 2024).

### 4.3 Interferon

IFN family is a group of cytokines that play a central role in resisting various infections and pathological processes. The expression of IFN-α and IFN-β (Type I interferons) may be defective in patients with neutrophilic asthma, which could be associated with reduced viral clearance capacity during acute exacerbations of asthma (da Silva et al., 2017). However, in patients with eosinophilic asthma, the levels of interferons in sputum cells are comparable to those in healthy individuals (da Silva et al., 2017). Impaired production of IFN makes asthma patients prone to viruses infections, and with uncontrolled type 2 immunity, it promotes AHR and inflammation, which may lead to the exacerbation of asthma (Rich et al., 2020). Notably, the production of type I interferons is critically regulated by interferon regulatory factor 7 (IRF7) (Ning et al., 2011). IRF-7 positively regulates the function of ILC2s via the transcription factor Bcl11b, and this regulation is independent of type I interferon signaling pathway (He et al., 2019). 2′-5′-oligoadenylate synthetase-like protein (OASL), a negative regulator of type I interferon, suppressed the type I IFN production from lung DCs induced by influenza virus, thereby protecting the function of lung ILC2s to promote IAV-induced airway inflammation and AHR (Chang et al., 2022). IFN-γ, a Type II interferon, is a cytokine secreted by Th1 cells which plays a crucial role in the immunomodulation of asthma. IFN-γ inhibits ILC2 proliferation and IL-13 expression *in vivo* and *in vitro*, thereby alleviating RV-induced mucous metaplasia, and its deficiency in the production in immature mice may lead to the development of asthma-like phenotypes after early RV infection (Han et al., 2017). IFN-λ (Type III interferons) are mainly produced in respiratory and intestinal epithelial cells and play an important role in combating viral invasion. Furthermore, IFN-λ can limit the decrease TSLP and IL-33 production, providing evidence for a protective role in asthma exacerbations (Won et al., 2019). However, the induction of IFN-λ in response to RV infection is impaired in asthmatic primary bronchial epithelial cells and alveolar macrophages, a phenomenon closely linked to the severity of asthma exacerbation resulting from RV infection (Contoli et al., 2006).

### 4.4 TGF-β

TGF-β is a pleiotropic cytokine, predominantly secreted by a variety of cell types, including airway epithelial cells, eosinophils, alveolar macrophages, alveolar epithelial cells, and fibroblasts, affecting cell growth, apoptosis, differentiation, migration, and the production of extracellular matrix (Saito et al., 2018). TGF-β plays a dual role in the pathogenesis of asthma, influencing both immune responses and airway remodeling (Morikawa et al., 2016). In viral infections and asthma, TGF-β seems to protect asthma patients and reduce viral susceptibility. After acute asthma, the strong anti-inflammatory TGF-β response can temporarily induce protection against influenza virus-induced immunopathology in the host, possibly by TGF-β1 maintaining overall lung integrity through directly suppressing the production of cytokines by various immune cells (Furuya et al., 2015a). In addition to reduced viral susceptibility in asthma, TGF-β plays a opposite role in the process of virus-induced exacerbation of asthma. Glucocorticoids (GCs) are frequently employed in the treatment of asthma. However, infections from respiratory viruses, including RSV, RV, or IAV, can result in elevated expression and activity of TGF-β in airway epithelial cells. This may interfere with the anti-inflammatory effects of GCs, potentially worsening the condition in asthma patients (Xia et al., 2017a).

## 5 Conclusion and perspectives

Asthma is a heterogeneous disease characterized by airway remodeling, AHR, and chronic airway inflammation, influenced by a variety of factors. It cannot be completely cured clinically at present but can only be managed with medication to improve the quality of life. Although allergic asthma is a common type, non-allergic asthma accounts for half of asthma cases, and its etiology is complex with unclear mechanisms, thus requiring attention. Pathogen infection is an important cause of non-allergic asthma, especially viral infections. RV and RSV are the main pathogens that trigger asthma in children, and the possible mechanism is that the bronchiolitis they cause leads to AHR, thereby triggering asthma. Furthermore, asthma patients are more susceptible to viruses, which can lead to the deterioration of asthma after infection, thereby causing recurrent asthma attacks.

The pathogenesis of asthma and viral infections is extremely complex but innate immune cells and cytokines play a key role in the development of both viral infections and asthma. This review aims to discuss the intricate network regulation from the perspective of innate immune cells and cytokines in three dimensions: virus-induced asthma exacerbation, increased susceptibility of asthmatic patients to viruses, and the worsening of asthma due to viral infections.

**Figure 1:**
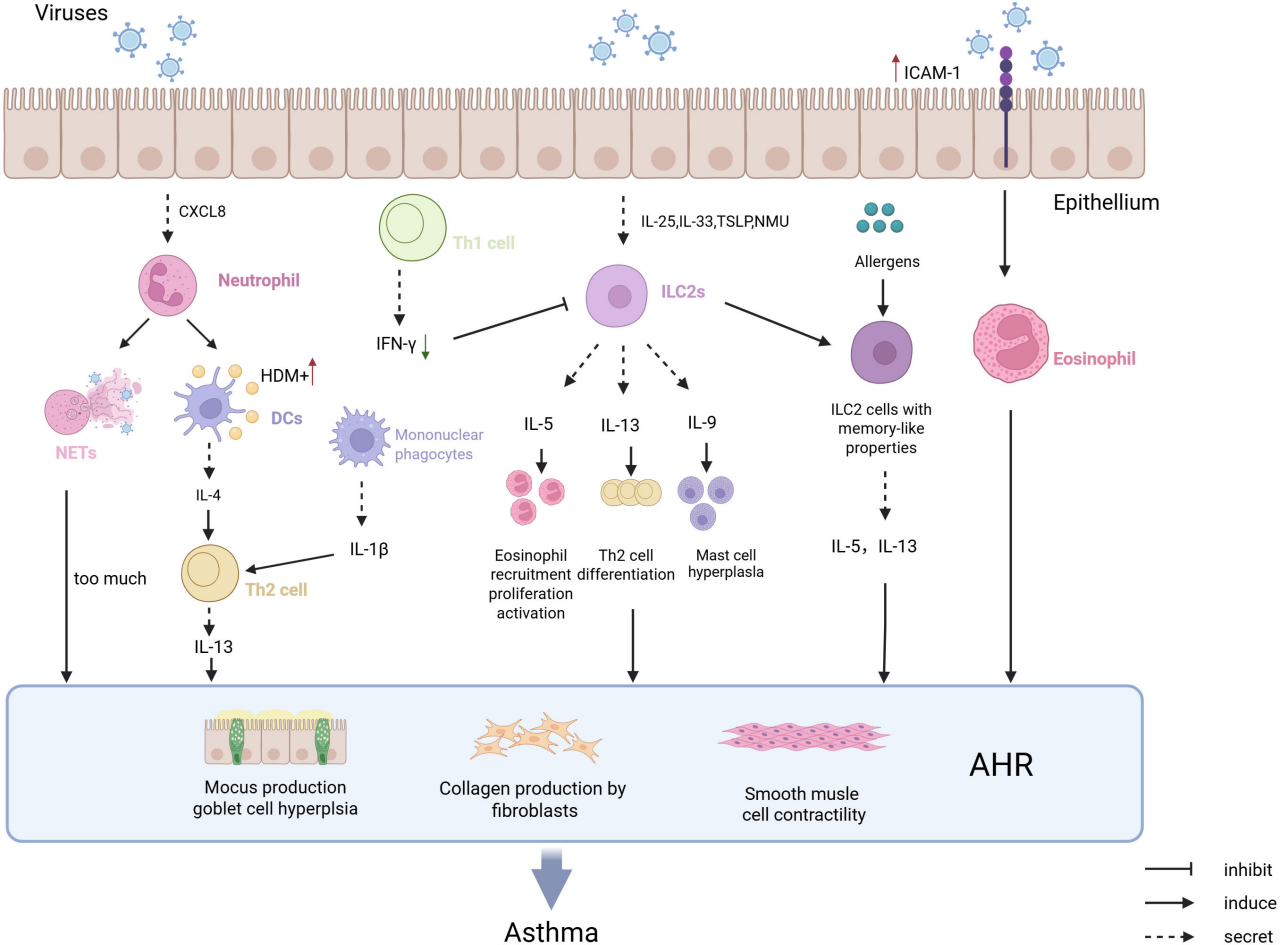
The pathological mechanism of virus infection-induced asthma attack. Epithelial cells infected by viruses secrete CXCL8, which chemoattracts neutrophils to form NETs. This process enhances DCs’ recognition of HDM and promotes IL-4 secretion, driving Th2-mediated type 2 immune responses that may trigger asthma. Viral infection of epithelial cells also leads to the secretion of TSLP and NMU, which chemoattract ILC2 cells and promote the production of type 2 cytokines, while generating memory-like ILC2 cells that increase the capture of allergens and promote the occurrence of asthma. Viral infection of epithelial cells upregulates the expression of ICAM-1, which promotes the adhesion of eosinophils and facilitates the development of asthma. Increased IFN-γ after RV infection can alleviate the onset of asthma symptoms and prevent the occurrence of asthma.

Viral infections trigger Th2-type immune responses by activating innate immune cells and releasing pro-inflammatory cytokines, serving as a key trigger for asthma attacks. (**Figure 1**). Respiratory syncytial virus (RSV) and rhinovirus (RV) infections activate airway epithelial cells via pattern recognition receptors (PRRs), leading to the release of alarmins such as IL-33 and TSLP (Boonpiyathad et al., 2019). These cytokines directly activate ILC2s and DCs, resulting in the secretion of cytokines like IL-5 and IL-13, which subsequently cause eosinophil infiltration into the airways, mucus hypersecretion, and airway hyperresponsiveness (AHR) (Lambrecht and Hammad, 2012; Stier et al., 2016). Moreover, RV infection through the TLR3/IL-33 pathway induces epithelial cells to express ICAM-1 and CDHR3 (RV-C specific receptor), not only enhancing viral adhesion but also binding to LFA-1/Mac-1 on eosinophils, thus increasing their adhesion capability and aggravating airway inflammation (Wardlaw et al., 1994; Bochkov et al., 2015). RV infection promotes the release of CXCL8, IP10, and CCL5 through the PRR-mediated NF-κB pathway, recruiting neutrophils and eosinophils (Herbert et al., 2017; Muehling et al., 2022). The formation of uncontrolled NETs by recruited neutrophils exacerbates airway remodeling and mucus hypersecretion while facilitating DC capture of HDM (Toussaint et al., 2017) (Radermecker et al., 2019). However, different viral subtypes have distinct pathogenic mechanisms: RV-C infects airway epithelium via CDHR3 receptor, closely associated with severe asthma attacks in children, whereas RV-A/B depends on ICAM-1, more related to asthma deterioration in adults (Bochkov et al., 2015). It is worth noting that early RSV infection-induced high expression of CCL5 correlates with long-term development of childhood asthma, though the causality remains unclear (Rosas-Salazar et al., 2023). However, whether viral-induced Th2 responses depend on allergen co-exposure remains controversial. Some studies show that pure RV infection can induce asthma-like inflammation without allergens, but allergic status significantly enhances viral pathogenicity in clinical cohorts.

**Figure 2:**
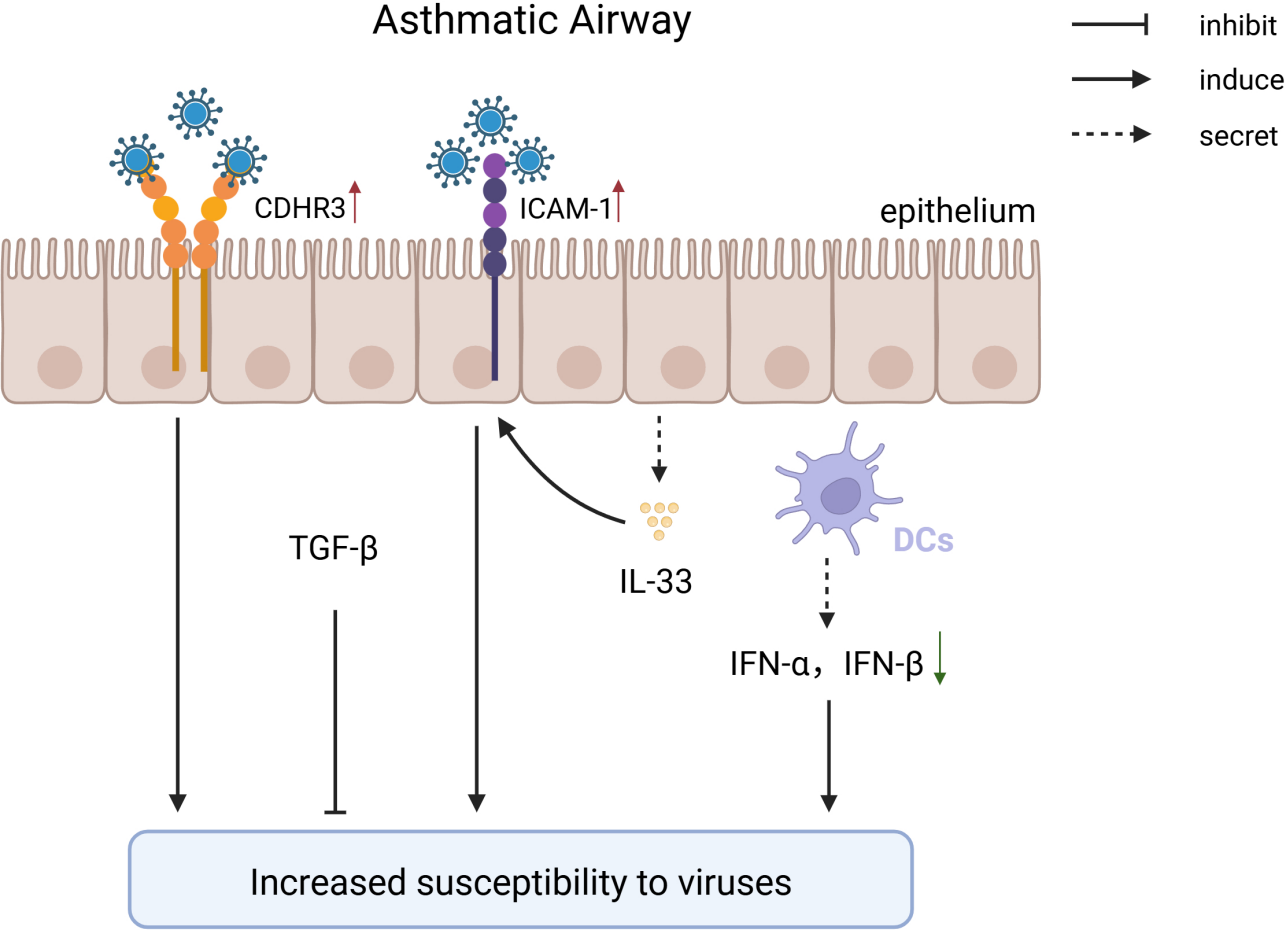
The pathological mechanism of increased susceptibility to viruses after being diagnosed with asthma. In individuals with asthma, genetic variations in the CDHR3 gene drive persistent overexpression of this receptor, thereby enhancing the binding of RV-C to airway epithelial cells and facilitating viral invasion. After infecting epithelial cells, RV-A/B promotes the expression of ICAM, facilitating viral entry; patients with asthma have a deficiency in type I interferon, leading to a reduced type I antiviral response, which increases susceptibility to viruses; TGF maintains lung stability through its powerful anti-inflammatory effects.

Asthmatic patients exhibit multiple defects in airway epithelium and innate immune cells, weakening antiviral capabilities and forming a vicious cycle of “increased viral susceptibility - asthma deterioration.” (**Figure 2**). Asthmatics have reduced numbers of cDC1, leading to insufficient viral antigen presentation and increased viral replication (Cameron et al., 2022). Meanwhile, pDCs express FcϵRIα at high levels, impairing TLR7/9 signaling pathways and inhibiting type I IFN secretion, causing higher viral loads post-RV infection compared to healthy individuals (Xi and Upham, 2020). Abnormal expression of viral receptors such as ICAM-1 in epithelial cells and MCs of asthma patients enhances viral adhesion and replication (Nakagome and Nagata, 2018). Interestingly, TGF-β plays a dual role in this context. In the researches about the susceptibility to a lethal influenza virus challenge and influenza virus-*S. pneumoniae* coinfection, TGF plays a protective role possibly through its strong anti-inflammatory response (Furuya et al., 2015b) (Roberts et al., 2019). Nevertheless, TGF-β plays a opposite role in the process of virus-induced exacerbation of asthma as its high expression weakens the anti-inflammatory effects of glucocorticoids (GCs), leading to treatment resistance (Xia et al., 2017b).

**Figure 3:**
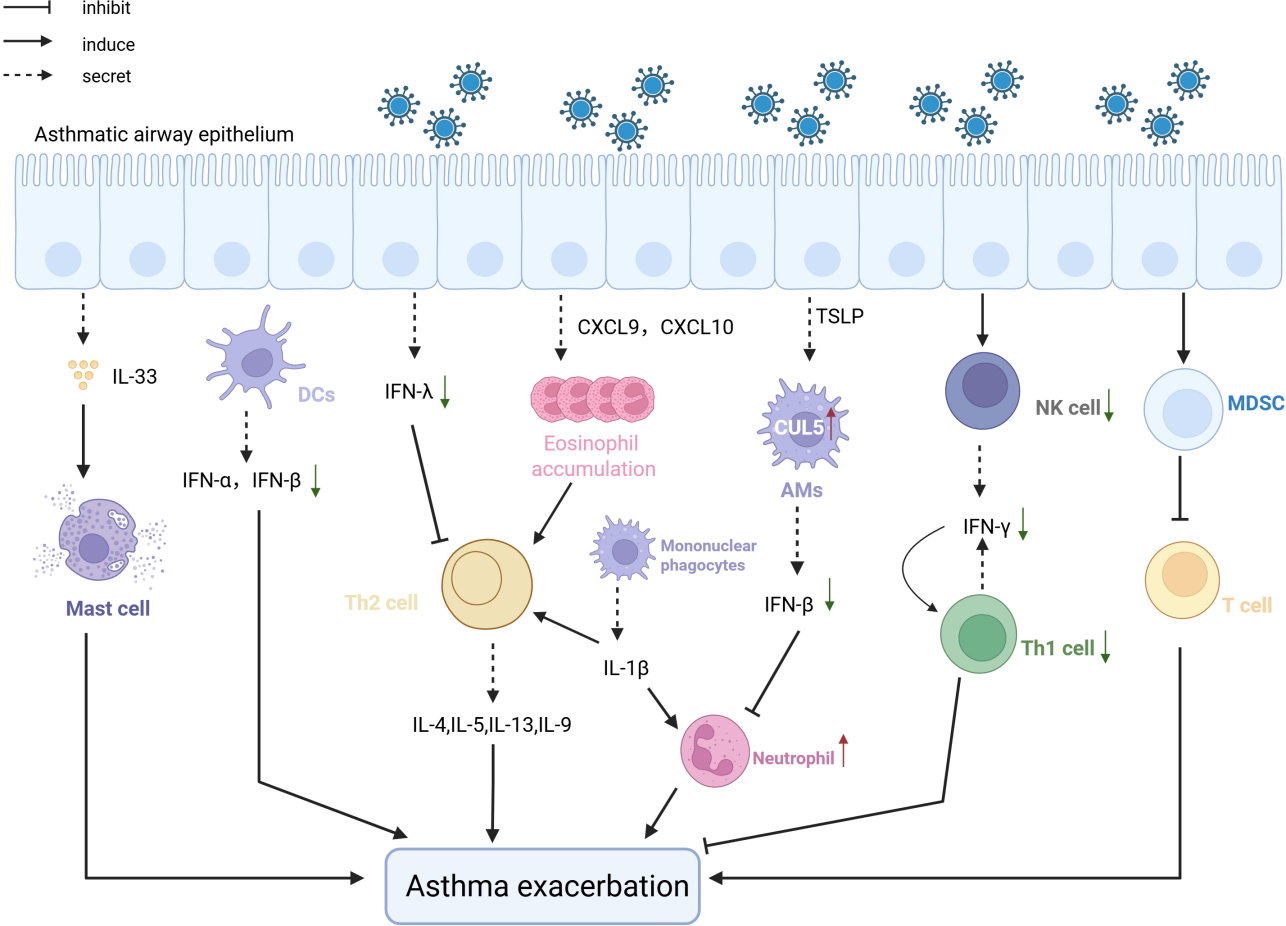
The pathological mechanism of the exacerbation of asthma Viral infection after viral infection induces asthma exacerbation. After infecting epithelial cells, viruses attract a large number of eosinophils through the CXCL-9/IP-10 -CXCR3 chemokine axis, which exacerbates asthma. Pre-existing allergic damage induces CUL5 expression, impairing antiviral immunity and promoting neutrophilic inflammation, leading to asthma worsening. NK cells and MDCK cells, following viral infection, respectively suppress asthma exacerbation by promoting antiviral Th1 responses and exerting immunosuppressive effects. Defects in type 1 and 3 interferons also lead to the worsening of asthma symptoms post-viral infection, due to uncontrolled type 2 immunity.

Viral infections exacerbate asthma acute deterioration by intensifying innate immune imbalance and inducing activation of memory immune cells. (**Figure 3**). RSV infection induces alveolar macrophages to secrete MMP-12, enhancing AHR and inflammatory cell infiltration (Makino et al., 2021). IAV infection activates macrophages via the NLRP3 inflammasome, releasing IL-33 that activates natural helper cells producing substantial IL-13 (Chang et al., 2011). Additionally, mILC2s differentiate after viral infections and reside in the gut lamina propria via CCR9/CCL25, rapidly migrating to the lungs upon re-infection through S1P/S1PR1 signaling, secreting IL-13 to amplify Th2 inflammation and induce steroid resistance (van der Ploeg et al., 2021; Bao et al., 2024). Unlike these cells, NK cells and MDSCs can alleviate asthma symptoms by secreting IFN-γ to enhance antiviral Th1 immune responses and exerting immunosuppressive effects to reduce T-cell activation, respectively, although clinical evidence is still required (Hegde et al., 2021) (van Geffen et al., 2024). Additionally, lack of type I and III interferons in asthmatic patients leads to uncontrolled type 2 immune responses following viral infections, thereby exacerbating asthma conditions (Rich et al., 2020) (Won et al., 2019).

In summary, innate immune cells and cytokines form a multidimensional network in the interaction between viral infections and asthma. Deciphering these mechanisms and targeting critical nodes such as TSLP, IL-33 and NETs, or restoring IFN responses, could provide new directions for precision therapy aimed at breaking the vicious cycle of “induction-susceptibility-exacerbation.” Current clinical strategies for asthma include targeting specific cytokines, such as Monoclonal antibodies targeting the eosinophilic inflammation pathway (IL-5R and IL-5), including mepolizumab, reslizumab, and benralizumab, are effective and safe for severe eosinophilic asthma (Principe et al., 2021). Otherwise, the anti-TSLP monoclonal antibody tezepelumab has shown potential in clinical trials to reduce asthma exacerbations and improve lung function (Corren et al., 2023). The treatment of asthma with viral infections can also be targeted at relevant immune cells and cytokines, and this review provides the direction for clinical treatment. In addition to these findings, the study highlighted the significant role of vaccines in the management of asthma exacerbations triggered by viral infections. Significant progress has been made in developing vaccines for respiratory syncytial virus (RSV). The US FDA has approved the first RSV vaccine for adults aged ≥60 years (Payne et al., 2024) and another for pregnant women to protect newborns through maternal antibodies (Zar et al., 2024). Meanwhile, influenza vaccination effectively reduces the risk of influenza infection in asthma patients, decreasing exacerbation frequency and severity (Kildegaard et al., 2023). Inactivated influenza vaccines are particularly preferred, especially in children under 5 years (Sokolow et al., 2022). However, no specific vaccine exists for rhinovirus (Bochkov et al., 2023), which is another common trigger of asthma exacerbations.

While this review endeavors to elucidate the complex interplay between viral infections and asthma through the lens of innate immunity, several critical limitations and unresolved questions must be acknowledged. Presently, the association between viral infections and nonallergic asthma is predominantly supported by epidemiological evidence, with a paucity of understanding regarding the mechanisms underlying the Th1/Th17 - dominated responses triggered by viruses, as opposed to Th2 polarization. Although NETs have been implicated in virus - induced airway remodeling, the precise viral ligands driving NETosis and their interactions with PRRs, such as TLR3 or NLRP3, in the context of nonallergic asthma remain to be clarified. Additionally, the role of epithelial - mesenchymal transition EMT following viral infections in nonallergic asthma, a process associated with airway fibrosis, has not been thoroughly investigated. The mechanisms by which viruses remodel epithelial cell fate through TGF - β or Wnt signaling pathways are also not well - understood. Furthermore, the metabolic reprogramming of innate immune cells during viral infections, which is essential for immune cell function, has not been fully explored in asthma, particularly in nonallergic subtypes.

In current clinical strategies for asthma management, targeted biologic agents and vaccines have demonstrated significant efficacy in reducing exacerbation risks and improving pulmonary function. However, their widespread implementation faces multifaceted challenges. The economic burden remains a primary barrier, as annual costs for biologic therapies often exceed tens of thousands of dollars. This imposes substantial out-of-pocket expenses even in regions with insurance coverage, while high prices severely limit accessibility in low- and middle-income countries. Although novel vaccines such as RSV vaccines have been approved, cost-related barriers hinder their global rollout. Influenza vaccines, despite being relatively affordable, still require government subsidies to ensure equitable access. Accessibility challenges include the dependence of biologics on cold chain transportation and specialized medical resources. Remote areas frequently experience treatment delays or drug inefficacy due to unstable power supplies and inadequate infrastructure in primary care settings. Additionally, inequitable vaccine distribution exacerbates disparities. Patient-specific factors, such as poor adherence and the lack of precision diagnostic tools (e.g., eosinophil level quantification and IL-4R gene polymorphism testing), further compromise therapeutic outcomes. For instance, real-world evidence indicates that biologics reduce hospitalization risk by 56%, yet efficacy variability reaches 60%. This underscores the need for phenotype-driven approaches (Panettieri et al., 2025).
